# P-2101. Rapid Point-of-Care Hepatitis C RNA Test-to-Treat in Persons Experiencing Homelessness through Street Based Initiatives in Detroit, Michigan

**DOI:** 10.1093/ofid/ofaf695.2265

**Published:** 2026-01-11

**Authors:** Yasmeen Mann, Kyle G Crooker, Mariia Numi, Michael Garcia, Brandon Ho, Richard Bryce, Marcus Zervos, Carl P Wilson, Shaina Shetty, Seema Joshi

**Affiliations:** Henry Ford Hospital, ON, Canada; Henry Ford Hospital, ON, Canada; Henry Ford Hospital, ON, Canada; Henry Ford Hospital, ON, Canada; Henry Ford Hospital, ON, Canada; Henry Ford Hospital, ON, Canada; Henry Ford Hospital, ON, Canada; Henry Ford Health, Detroit, Michigan; CHASS Center, Detroit, Michigan; Henry Ford Hospital, ON, Canada

## Abstract

**Background:**

Persons experiencing homelessness (PEH) are diagnosed with higher rates of untreated Hepatitis C Virus infection (HCV) than housed individuals, due to increased risk factors for acquiring HCV and barriers in access to care. We describe a street-based initiative for PEH using the Cepheid point-of-care (POC) HCV RNA test (Xpert® HCV) for access to same day diagnosis of HCV and simplified treatment.

**Figure:**
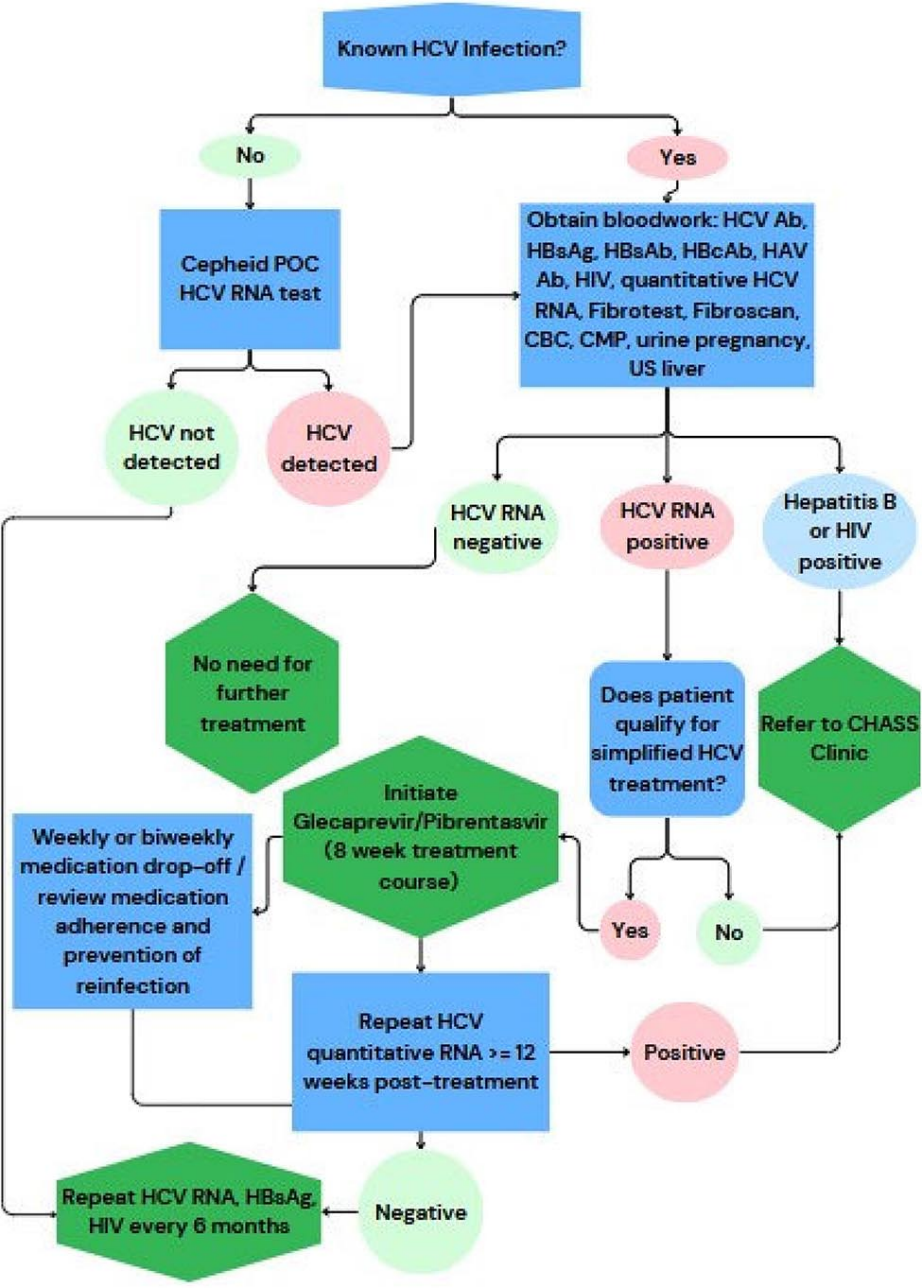
Protocol for Testing

**Figure:**
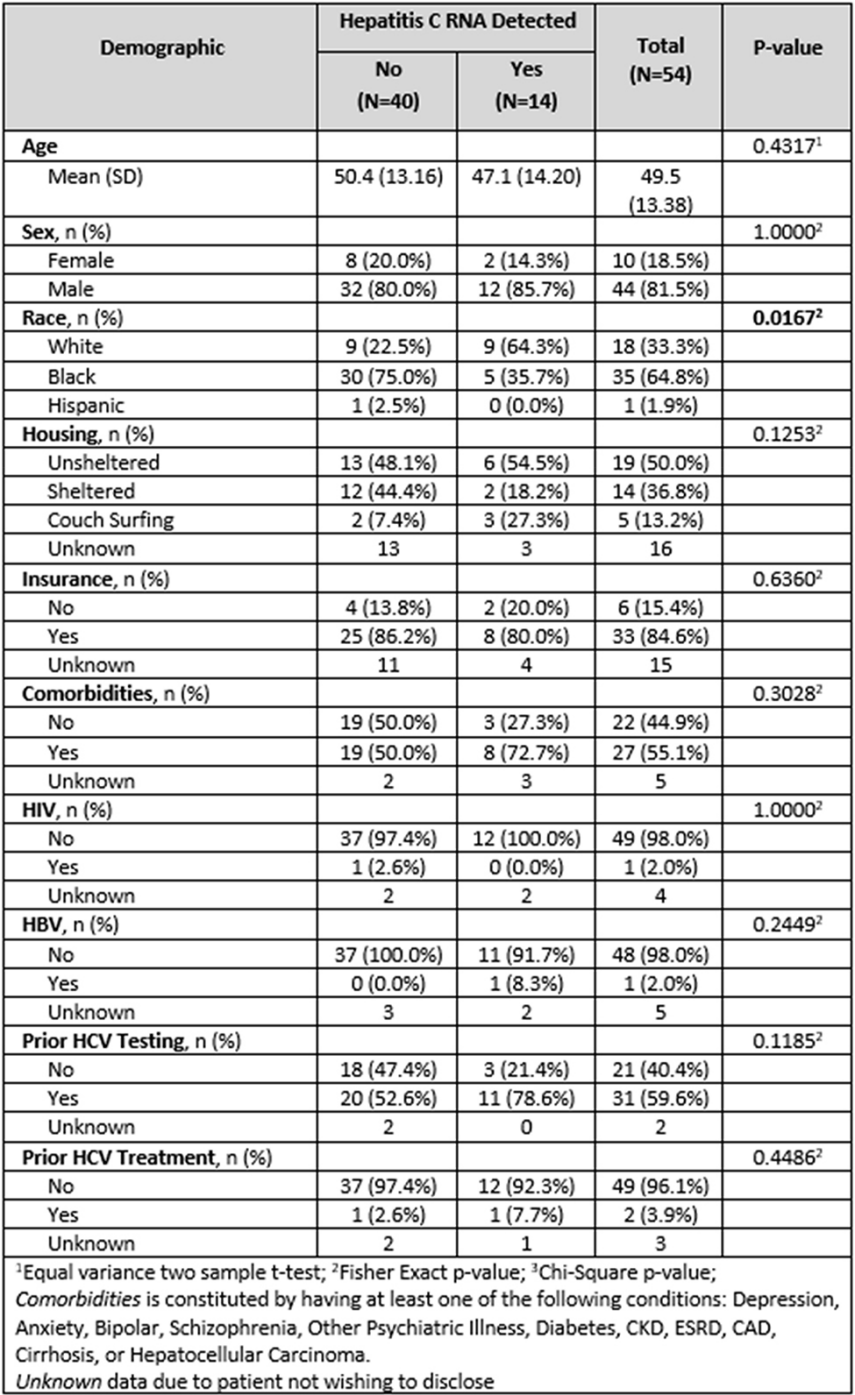
Demographics of HCV Positive and Negative Participants

**Methods:**

In collaboration with Henry Ford Hospital, Community Health and Social Services Center (CHASS), and local Street Medicine Organizations, a group of medical providers provided weekly street-based POC HCV RNA with same day confirmation (Figure 1). If RNA positive, participants underwent confirmatory bloodwork and testing to evaluate eligibility for direct acting antiviral therapy. Weekly or biweekly medication supply was provided to patients with follow up to assess adherence. Participants were offered $10.00 gift cards to a local grocery store for testing and weekly medication adherence. Demographics and risk factors were compared retrospectively via Chi-Squared test or Fisher’s Exact test.

**Figure:**
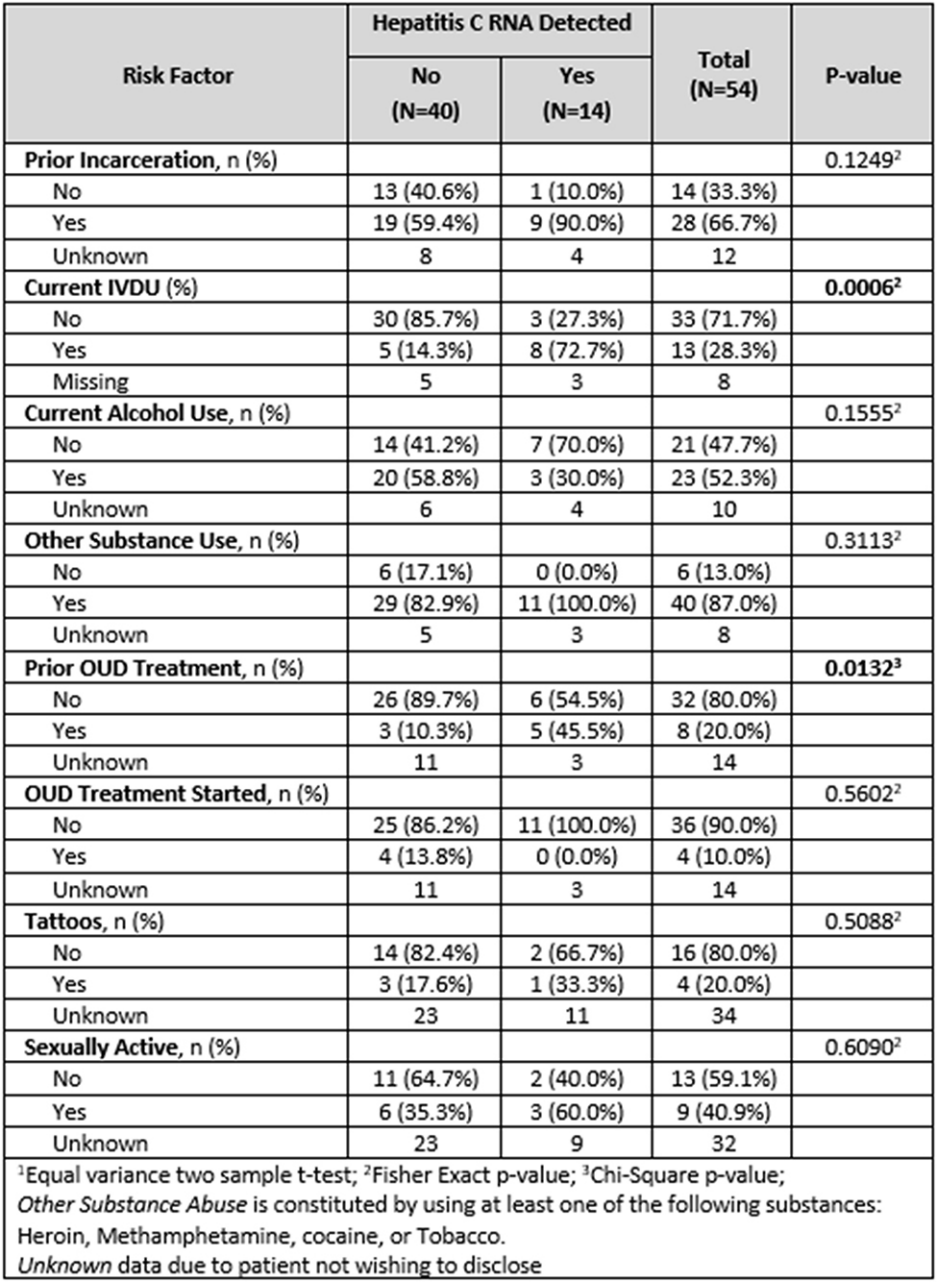
Risk Factors for HCV Positive and Negative Participants

**Results:**

Testing occurred November 2024-present with weekly street-based runs. 54 patients were tested with Xpert® HCV (Tables 1 & 2). HCV RNA was confirmed in 14 individuals. 10 tested positive on the street and 4 tested positive at shelter-based runs. 3 patients were successfully treated with HCV RNA undetectable, 3 are undergoing treatment, 1 started treatment but later died from causes unrelated to HCV, 6 patients are pending confirmatory bloodwork but have been notified of positive results, and 1 patient did not clear infection due to suspected reinfection from needle sharing intravenous drug use (IVDU).

A significant proportion of patients overall were white compared to other races (64.3% versus 35.7%, p=0.0167). Current IVDU was significantly higher for patients testing positive (72.7% versus 14.3%, p=0.0006). Previous opioid use disorder (OUD) treatment was significantly higher for patients testing positive (45.5% versus 10.3%, p=0.0132).

**Conclusion:**

This study demonstrates the importance of using novel interventions such as Cepheid POC HCV RNA testing in a street-based initiative to reduce barriers in access to care for HCV testing and treatment of PEH.

**Disclosures:**

Marcus Zervos, MD, merck: Honoraria Seema Joshi, MD, Cephied: Grant/Research Support

